# Fishing for ancestry

**DOI:** 10.7554/eLife.39524

**Published:** 2018-08-02

**Authors:** Hannah Brunsdon, E Elizabeth Patton

**Affiliations:** 1 MRC Human Genetics Unit & Cancer Research UK Edinburgh CentreMRC Institute of Genetics and Molecular Medicine, University of EdinburghEdinburghUnited Kingdom

**Keywords:** epidermal appendage, scales, skin, patterning, morphogenesis, signaling, Zebrafish

## Abstract

The same genes and signalling pathways control the formation of skin appendages in both fish and land animals.

**Related research article** Aman AJ, Fulbright AN, Parichy DM. 2018. Wnt/β-catenin regulates an ancient signaling network during zebrafish scale development. *eLife* **7**:e37001. doi: 10.7554/eLife.37001

The tough scales found on alligators, the colourful feathers of peacocks and the thick fur of polar bears are all examples of skin appendages – self-contained mini-organs that form a barrier between an organism and its environment (Chuong et al., 2013). These skin appendages are essential for providing protection, retaining heat, sensing movement, attracting mates and, in some animals, flying. How these immensely complex and diverse structures have evolved, and how they develop, has fascinated researchers for decades.

Due to the sheer variety of skin appendages in both living and fossilised animals, it is still unclear whether they evolved independently or from a common ancestor (Dhouailly et al., 2017). However, as some of the earliest processes involved in the development of feathers and hair are very similar, the basic genetic mechanisms underlying their formation could have shared evolutionary origins (Musser et al., 2015). Indeed, researchers recently found that the cell signalling events involved in the development of scales in reptiles and the events regulating the patterning of appendages in birds and mammals have many similarities (Di-Poï and Milinkovitch, 2016). For example, slightly altering gene expression in reptile scales or the scaly foot of a chicken can lead to the formation of feather buds and primitive feathers respectively, akin to those found in dinosaurs (Wu et al., 2018).

Therefore, it seems likely that the skin appendages of reptiles, birds and mammals – collectively known as amniotes – have a shared evolutionary origin. Fish scales appear to be different in that they are made of bone instead of keratin, and also derive from a different layer of skin. However, is it possible that amniotes and fish share a common, ancestral skin appendage that existed even further back in time than was previously thought? Now, in eLife, Andrew Aman, Alexis Fulbright and David Parichy of the University of Virginia report the results of experiments on zebrafish which suggest that this might be the case (Aman et al., 2018).

Through beautifully detailed live imaging of developing scales and the manipulation of key signalling pathways during their development, Aman et al. were able to study how zebrafish scales form and arrange themselves correctly on the skin. The first hint that appendage patterning in fish is similar to that of amniotes came from close observation of scale development, which occurred sequentially in a hexagonal grid pattern to cover the fish, a patterning strategy shared with the land animals.

Aman et al. then investigated whether the cell signalling mechanisms underpinning these morphological events might also be conserved at the molecular level. In all amniote species investigated so far, the earliest signalling event in appendage formation is the activation of the Wnt signalling pathway, which regulates many aspects of cell development and behaviour. Using genetically modified fish to highlight Wnt-signalling cells, it was possible to identify clusters of cells congregating at sites that would later form scales. Aman et al. showed that Wnt signalling and the subsequent activation of another signalling pathway, the Eda pathway, were both necessary for the scales to develop, and that blocking either pathway caused a complete loss of scale formation. This skin appendage loss is consistent with the one observed in fish, bearded lizards, chicks, mice and humans with defective Eda signalling (Harris et al., 2008; Sennett and Rendl, 2012; Drew et al., 2007; Di-Poï and Milinkovitch, 2016).

Next, Aman et al. disrupted combinations of these and other signalling systems known to be involved in the development of skin appendages in amniotes, to expand our knowledge of appendage development. These experiments revealed that, similar to birds and mammals, another signalling system that is important for the development and repair of cells, the fibroblast growth factor system, was required for the scales to differentiate further (Mandler and Neubüser, 2004; Huh et al., 2013). Moreover, the researchers discovered a new role for another well-known signalling system, the Sonic hedgehog system, which instructed the top layer of the skin to fold over the scales, similar to the skin movements that occur as hair follicles develop (St-Jacques et al., 1998). These similarities hint at the existence of a single, ancient origin of appendage patterning in vertebrates (Figure 1).

**Figure 1.**
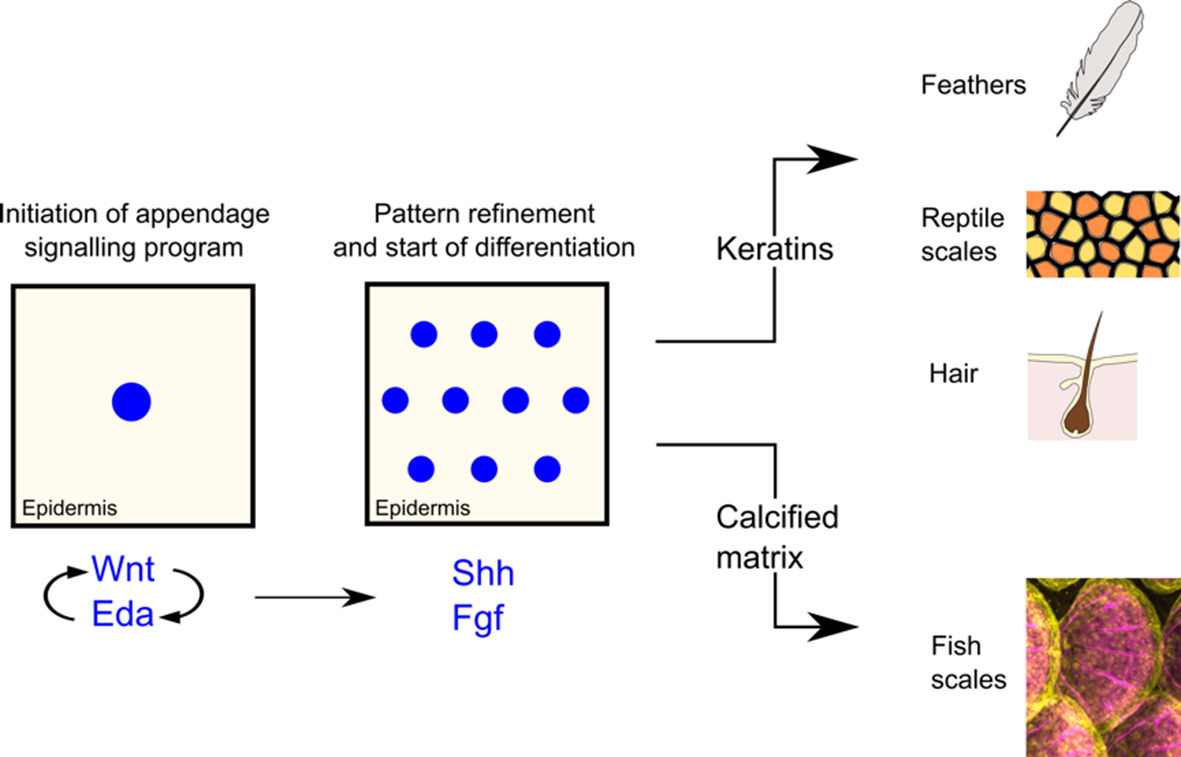
Simplified schematic illustrating the development of skin appendages. The development of skin appendages is initiated by the activation of the Wnt signalling pathway in the top layer of the skin (the epidermis), and this signal is refined and maintained by the Eda signalling pathway. Both systems activate two further pathways that are important for cell growth and development: fibroblast growth factor (Fgf) signalling is necessary to ensure that the patterning of the appendages happens correctly and that scales are able to form; the Sonic hedgehog pathway ensures that the epidermis folds properly over the scales. Fish scales are formed from bone, whereas feathers, reptile scales and hair are formed from keratin. However, over time a common ancestor may have stopped using bone and started using keratin to build skin appendages in amniotes.

Discovering such conserved gene regulatory networks in the zebrafish – an organism that is so readily amenable to high-resolution imaging and genetic manipulation – is very exciting. In amniotes, these fleeting events mainly occur in the womb or egg, which makes it difficult to collect stage-matched animals and use live imaging – a challenge in identifying key signalling processes (Sennett and Rendl, 2012).

If we could further untangle the contribution of various genes involved in the development of skin appendages, we might be able to apply this knowledge to tissue engineering (by, for example, regulating hair patterning on skin grafts). Also, by identifying new genes or functions of genes in appendage development, zebrafish could help us understand and ultimately treat skin disorders in humans.
